# Spurious-Mode Suppression and Efficient Design of LiTaO_3_ XBAR Filters Using a FEM–COM–ABC Framework

**DOI:** 10.3390/mi17101203

**Published:** 2026-10-18

**Authors:** Jiabao Tang, Zifan Miao, Yunjing Zhang, Xingli He

**Affiliations:** School of Electronic and Information Engineering, Soochow University, Suzhou 215006, China

**Keywords:** acoustic filters, artificial bee colony optimization, coupling-of-modes modeling, finite-element method, laterally excited bulk acoustic wave resonators, lithium tantalate, spurious-mode suppression

## Abstract

Laterally excited bulk acoustic wave resonators (XBARs) on lithium tantalate (LiTaO_3_, LT) are promising for wideband radio-frequency filters, but spurious modes and costly full-wave optimization hinder development. Limited coupling-bandwidth flexibility in commercial LT cuts motivates exploring non-standard orientations. We present a numerical framework integrating the finite-element method (FEM), coupling-of-modes (COM) theory, and artificial bee colony (ABC) optimization, emphasizing non-commercial 120° YX-LT. Hierarchical FEM screens orientation and periodic-cell response and then resolves finite aperture, busbars, and membrane boundaries. Compared with rectangular boundaries, an irregular pentagonal membrane disrupts coherent edge reflections, suppressing in-band spurious glitches and slightly enhancing effective coupling. A thickness-dependent COM model calibrated across a broad SiO_2_ loading range reproduces FEM resonance and antiresonance frequencies within 1 MHz. The surrogate enables mixed-variable ABC optimization of finger-pair number, normalized aperture, and stage-specific wavelengths. Five-stage ladder filters are synthesized and verified by direct FEM reconstruction. Stage-dependent wavelengths improve simulated passband transmission and reduce ripple versus a common-wavelength baseline; COM–FEM stopband discrepancies indicate the need for full-wave verification. After calibration, ABC core search takes approximately 2.9 h without repeated full-wave solutions. This transferable framework provides a cost-effective pathway from full-wave physics to circuit-level filter synthesis on thin-film piezoelectric platforms.

## 1. Introduction

The evolution of fifth-generation (5G), Wi-Fi 6/7, and prospective sixth-generation (6G) systems requires radio-frequency front-end filters operating above 3 GHz with wide bandwidth, low insertion loss, strong rejection, and compact size. Conventional surface acoustic wave (SAW) filters face lithographic and propagation-loss constraints at high frequencies, whereas bulk acoustic wave (BAW) devices are often bandwidth-limited by moderate electromechanical coupling. Laterally excited bulk acoustic wave resonators (XBARs) address these limitations by using interdigital transducers (IDTs) to excite the thickness-dominated first-order antisymmetric Lamb wave (*A*_1_) or the plate shear wave (*SH*_0_) in a sub-μm piezoelectric plate [1]. Resonance is therefore governed mainly by plate thickness rather than by deeply scaled electrode pitch [2,3,4,5].

Most XBAR research has focused on single-crystalline LiNbO_3_ (LN). Early thin-plate studies demonstrated high phase velocity and large resonance–antiresonance separation [2,3]. A 5 GHz XBAR subsequently achieved approximately 25% coupling [4], and a Band-n79 ladder filter demonstrated more than 600 MHz bandwidth with 1.35 dB minimum insertion loss [5]. With an optimized LN crystal cut (e.g., 128° YX), the effective coupling of XBAR *k_t_*^2^ can exceed 46% [6]. Later studies showed that membrane thickness and electrode pitch determine the modal and capacitive operating point, while metal coverage, aperture, and finite boundaries affect coupling, quality (*Q*-) factor, and higher-order responses [7,8,9,10,11]. However, the same dispersion and anisotropy that enable wide bandwidth also promote *A*_1_ harmonics, transverse resonances, edge-reflected waves, and leakage.

Spurious mode suppression remains one of the critical challenges for XBARs. Spurious-suppression methods can be grouped by physical mechanism. Electrode-shape engineering disrupts the lateral periodicity that supports coherent parasitic modes: pentagon-spiral and arc-shaped electrodes redistribute reflection paths [12,13], while trapezoidal electrodes vary the local pitch to suppress discrete transverse responses [14]. Such designs directly modify transduction but may perturb the desired *A*_1_ mode or increase lithographic complexity. Boundary engineering instead controls waves leaving the active aperture. Grooves scatter lateral energy [15], reduced-gap reflectors improve confinement [16], and edge-narrowed IDTs modify boundary impedance while enhancing the *Q*-factor [17]. Scattering-hole arrays form distributed barriers [18], whereas external reflective arrays redirect useful energy toward the aperture [19]; both depend strongly on placement and phase matching. Other approaches reshape the electrode edge or vertical acoustic boundary. Arched-edge double electrodes and crescent busbars weaken coherent edge reflection and have achieved high figures of merit [20,21]. Solidly mounted *A*_1_ resonators and filters employ Bragg reflectors to reduce substrate leakage and improve mechanical support [22,23], although their multilayer stacks add thickness tolerances and interfaces. Thus, spurious suppression cannot be treated as an isolated geometric correction: it must be balanced against coupling, *Q*-factor, fabrication complexity, and filter-level ripple and rejection.

Compared with LiNbO_3_, lithium tantalate (LiTaO_3_, LT) offers a relatively unexplored alternative. Commercial SAW technology has established appropriate LT cuts because they combine low propagation loss, favorable temperature stability, restrained parasitic responses, and mature processing. Although LT is expected to provide lower XBAR coupling than LN, reduced loss, weaker unwanted-mode excitation, simpler suppression structures, and an established supply chain may offer a better overall compromise for selected bands. Composite-film research also indicates that piezoelectric configuration can tailor coupling [24]. Nevertheless, LT-XBAR studies remain scarce, and no accepted filter-design methodology has emerged. Our group has fabricated high-frequency XBAR resonators on 42° YX-cut LiTaO_3_ (42YX-LT) thin films, but commercially available thin-film LT substrates with this crystallographic orientation are unsuitable for developing high-frequency filters [25].

A further challenge is linking resonator physics to filter synthesis. Existing workflows typically use two- or three-dimensional finite-element method (FEM) simulations for modal analysis and then fit Butterworth–Van Dyke (BVD) or modified BVD circuits for ladder-network design [5,10,26,27]. The three-dimensional FEM becomes costly when optimizing many resonators and design variables. An analytically assisted FEM and GPU acceleration shorten simulations [28,29], but repeated network optimization still requires a compact model. Conversely, lumped BVD models reproduce admittance near resonance without explicitly representing distributed propagation, reflection, transduction, attenuation, or mode conversion in a finite IDT, especially one with irregular boundaries.

The coupling-of-modes (COM) formalism bridges full-wave analysis and network synthesis by representing forward and backward acoustic waves, reflection, and electrical excitation with physically interpretable parameters. A distributed COM model can preserve the propagation and boundary effects needed for irregular IDTs while remaining efficient for filter synthesis. Although COM is established for conventional SAW devices, XBAR research remains dominated by the FEM, dispersion analysis, and BVD fitting. The development of a COM model enabling accurate and efficient modeling of LN/LT-based XBARs is highly desirable.

**Figure 1:**
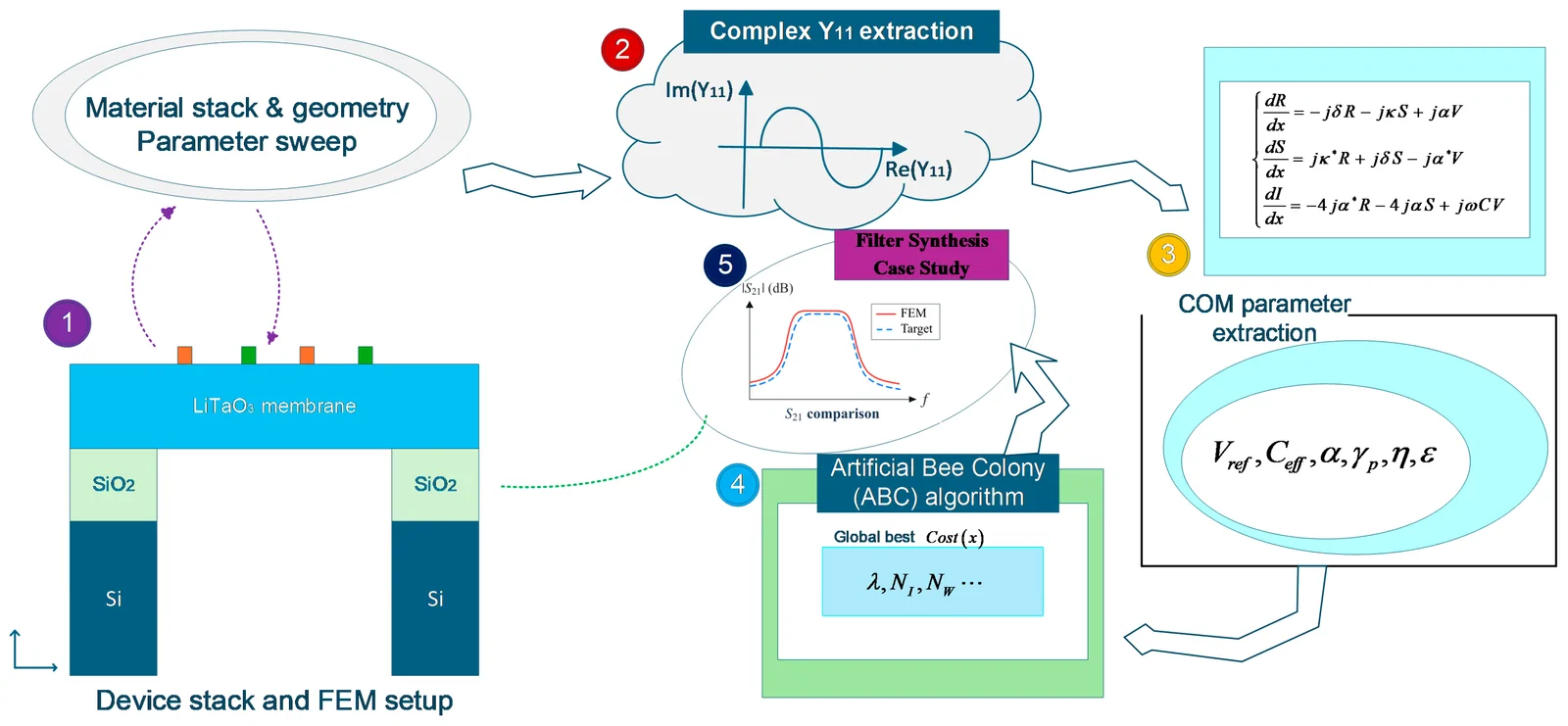
FEM/COM/ABC-assisted workflow for resonator characterization, filter synthesis, and direct FEM verification. The asterisk denotes complex conjugation in the COM equations; solid arrows show the main workflow, and dashed arrows show parameter-sweep and direct-FEM-verification links.

Building on the established FEM, COM, and artificial bee colony (ABC) optimization [30], this work develops a design framework for 120° YX-LiTaO_3_ XBAR filters (Figure 1). Its methodological contribution is a FEM-calibrated, thickness-dependent effective COM parameterization over 0–80 nm SiO_2_ loading, coupled to mixed-variable optimization of finger-pair number, normalized aperture, and resonator wavelength. This coupling enables filter synthesis without repeated full-wave solutions during the search. Numerical comparisons with matched active IDT areas show weaker spurious features near the main resonance for an irregular pentagonal layout while retaining the principal *A*_1_ response. For the investigated five-stage filters, allowing stage-specific wavelengths reduces passband ripple from 1.10 to 0.70 dB in cascades of FEM-derived resonator admittances.

## 2. Materials and Methods

### 2.1. FEM Simulation

**Figure 2:**
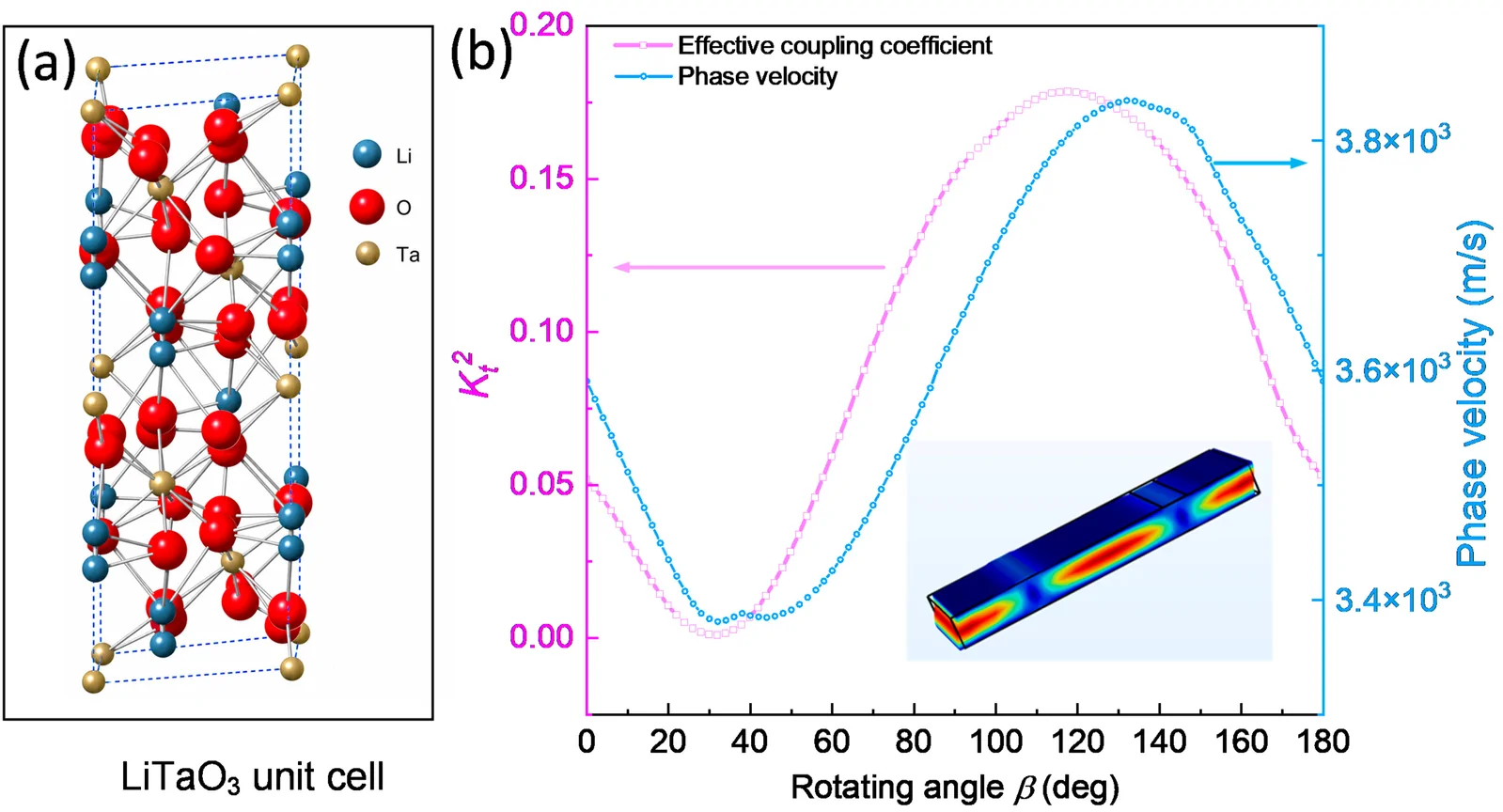
(**a**) 3D unit cell of LiTaO_3_ (space group R3c); (**b**) *k_t_*^2^ and *Vp* versus rotation angle *β* for Y-cut LiTaO_3_ XBARs.

LiTaO_3_ is a trigonal ferroelectric with strong piezoelectricity, low acoustic loss, and favorable chemical and thermal stability. Its elastic, dielectric, and piezoelectric tensors are anisotropic; therefore, the polarization, phase velocity, coupling, and modal spectrum depend on both the wafer cut and the in-plane propagation direction. Consequently, crystal orientation was treated as the first FEM design variable. The non-centrosymmetric R3c structure in Figure 2a produces spontaneous polarization along the *c*-axis. Under the lateral field of an IDT, the chosen tensor rotation couples this polarization to the thickness-dominated *A*_1_ mode. The rotation also alters the effective stiffness and thus the acoustic velocity, so coupling and velocity must be evaluated together.

The LT tensors were rotated using Euler angles (0°, *β*, 0°). For each angle, resonance and antiresonance frequencies, effective coupling *k_t_*^2^, and phase velocity *V_p_* were obtained from the electrical response. This sweep defines a material-level operating window before introducing electrode geometry and finite-boundary topology.

Figure 2b shows that Y-cut LT maintains a phase velocity suitable for multi-gigahertz operation and achieves a simulated coupling of about 18%. Coupling collapses near *β* = 30°, whereas *β* = 120° provides a useful compromise between velocity and *k_t_*^2^ and was selected for subsequent simulations. It is implemented as (0°, −30°, 0°) in the 3D COMSOL Multiphysics 6.4 convention and as (0°, −120°, 0°) in 2D. Keeping this convention fixed avoids unintended sign changes in the rotated tensor components.

**Figure 3:**
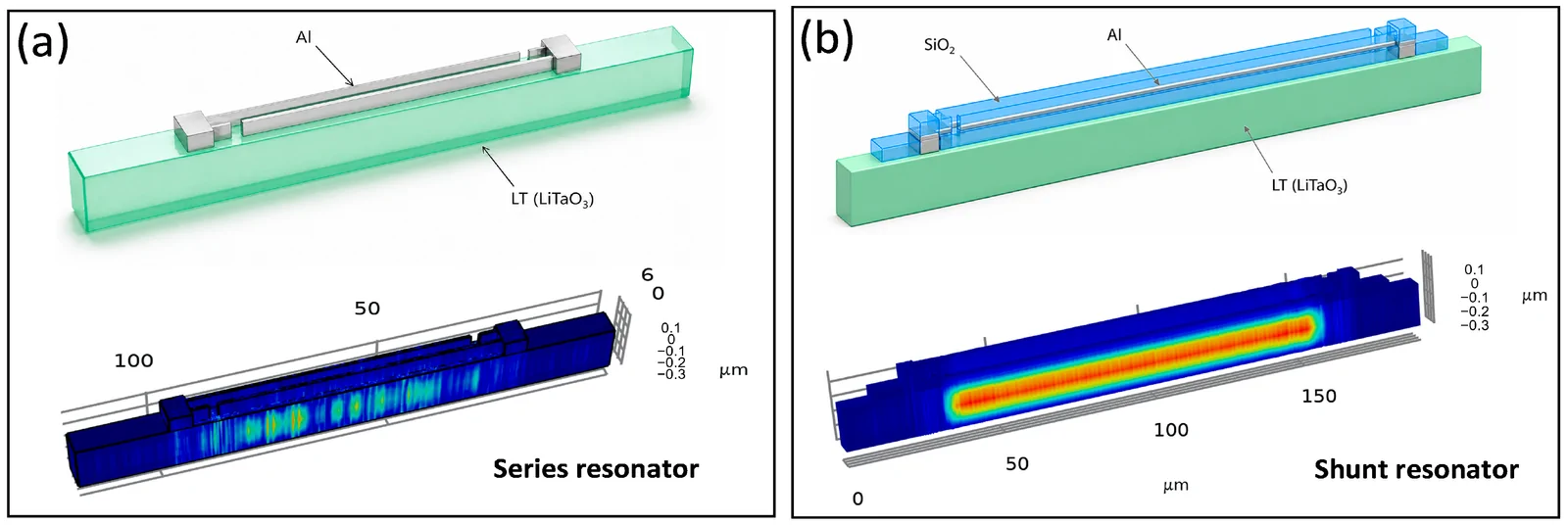
Images of single pair series (**a**) and shunt (**b**) components of the LT-XBAR.

A single-period model was then used to screen geometry efficiently. One IDT finger pair explicitly represented electrode mass, lateral field concentration, and stress through the LT plate (although commercial LiTaO_3_ thin films are usually available in thicknesses between 200 nm and 1 μm, we adopt a fixed thickness of 300 nm throughout this work). The series cell in Figure 3 uses Al electrodes on bare LT (the finger width of the Al electrodes was fixed at 500 nm, while the spacing between the electrodes could change). To generate the frequency offset between the series and shunt resonators, two approaches are available: local deposition of an SiO_2_ film (for the shunt branch) or selective thinning of the single-crystal piezoelectric film (for the series branch) [27,31]. The latter method is potentially advantageous for preserving the *Q*-factor of individual resonators in the XBAR filter. However, because of the challenges associated with ion etching and precise thickness control of LN or LT thin films, this work resorts to local SiO_2_ deposition on selected resonators to achieve the required frequency split. The shunt cell adds a SiO_2_ overlayer. This additive layer shifts the shunt response without locally etching the piezoelectric membrane.

Periodic boundaries were applied along the propagation direction, with low-reflecting conditions at the transverse ends. Alternating Al fingers served as the electrical terminals, and the remaining surfaces followed the suspended XBAR configuration. The mesh was refined through the LT plate and near the Al and SiO_2_ edges, where electric and stress gradients are largest. This unit-cell model enables rapid sweeps of wavelength, metal thickness, and dielectric loading. The deformation maps in Figure 3 are dominated by the *A*_1_ mode: lateral excitation generates a thickness-shear field whose displacement reverses across the plate. Energy remains concentrated beneath the overlap, confirming effective excitation. The SiO_2_ coating also alters localization, impedance, dispersion, and leakage, so it must be included in the later COM extraction as more than a simple frequency shift.

To separate lateral and thickness effects, the LT thickness was fixed at 300 nm and the aperture at 10*λ*. Wavelength or Al thickness was varied independently, while the other dimensions were held constant.

**Figure 4:**
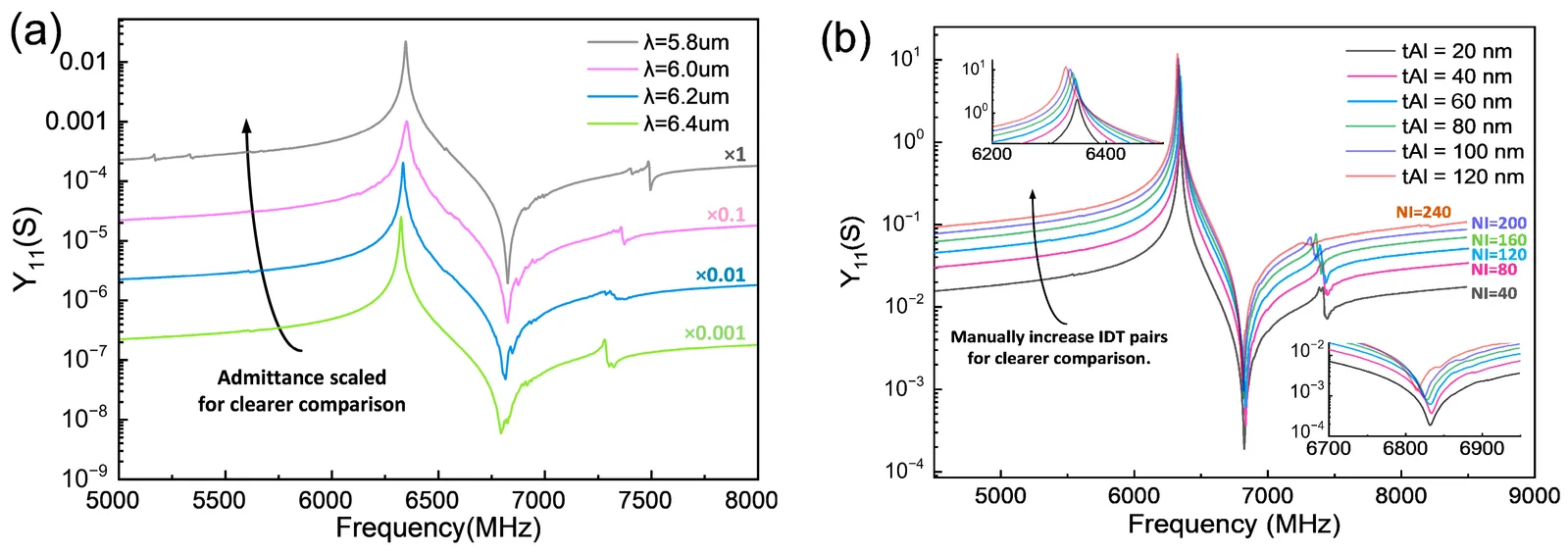
FEM-simulated LT-XBAR resonators’ responses employing different wavelengths (**a**) and Al electrode thicknesses (**b**). The thickness of LT is fixed at 300 nm, and the width of the aperture is 10*λ*.

Reducing wavelength *λ* from 6.4 to 5.8 micrometers produces only a small upward resonance shift in Figure 4a, and increasing Al thickness from 20 to 120 nm shifts the resonance by less than 30 MHz in Figure 4b. Thus, unlike a conventional SAW, the LT-XBAR frequency is set mainly by plate thickness. Pitch and metal thickness remain important because they tune capacitance, transduction, edge reflection, impedance, and spurious modes.

**Figure 5:**
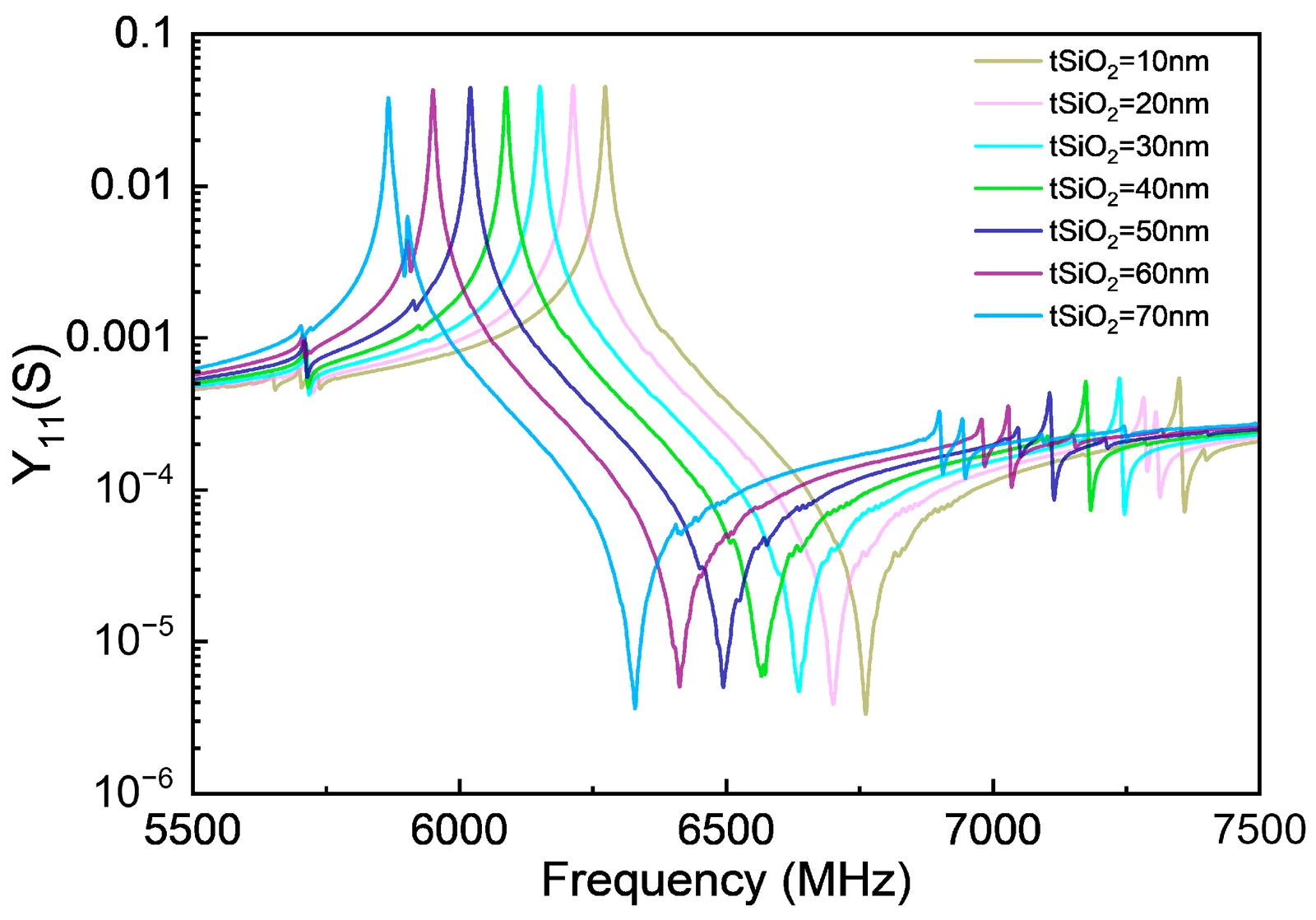
FEM-simulated responses of LT-XBAR shunt resonators with different SiO_2_ mass loadings. The width of the aperture is 15*λ*.

A ladder filter also requires a controlled offset between series and shunt resonators fabricated on the same LT film. Local LT thinning can raise the frequency but risks surface damage and thickness nonuniformity. The adopted process leaves the series branch uncoated and adds SiO_2_ to the shunt branch. Deposition thickness is easier to control and lowers the shunt resonance without altering the underlying LT. Figure 5 confirms a systematic downward shift as SiO_2_ thickness increases. The offset is nearly linear over the investigated range, allowing a target series–shunt separation to be translated into an initial coating thickness before a narrow FEM refinement. Capacitance, coupling, attenuation, and reflection are extracted simultaneously because all four vary with the overlayer.

The periodic cell cannot reproduce all practical resonant modes. A finite XBAR includes many fingers, busbars, electrode terminations, and a clamped, suspended-membrane perimeter. Waves leaving the active aperture can return from the edge and interfere with junction scattering, generating closely spaced parasitic peaks: the spurious modes. N. F. Naumenko discussed an optimization method based on BAW slowness-surface analysis to suppress spurious modes in XBARs by selecting LiNbO_3_ orientations (0°, 4–15°, 90°) and optimal plate thicknesses (0.05–0.1λ). The *A*_1_ mode is converted into a vertically propagating shear BAW, decoupled from spurious responses [32]. Nevertheless, the current analysis is confined to a two-dimensional framework and neglects the influence of layout configurations and electrode-pair arrangements on the overall response of full XBAR resonators with multiple IDT pairs. Unit-cell parameters therefore describe the local interaction region, while a complete device model is required to account for edge loss, ripple, etc. Herein, full three-dimensional resonators were constructed with their actual finger count, aperture, busbars, terminations, and membrane boundary. Admittance and surface deformation were computed across the main *A*_1_ response and neighboring modes using the same orientation and mesh criteria. The fields identify acoustic escape paths and distinguish genuine suppression from a mere displacement of a parasitic peak.

**Figure 6:**
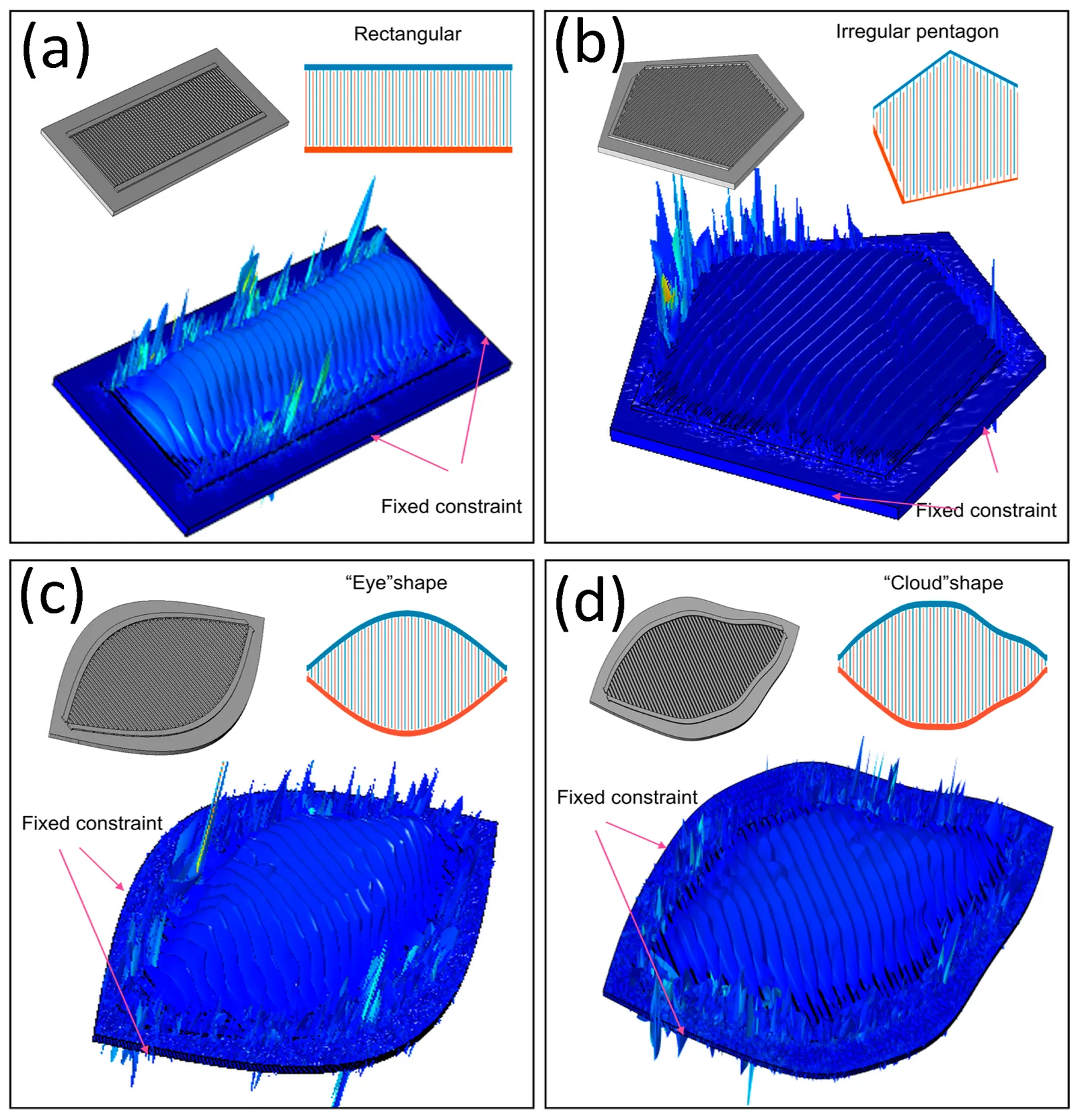
Complete models of LT-XBAR devices with (**a**) rectangular, (**b**) irregular pentagonal, (**c**) eye-shaped, and (**d**) cloud-shaped geometries.

Figure 6 compares rectangular, irregular pentagonal, eye-shaped, and cloud-shaped devices at *λ* = 5.8 μm, *N_I_* = 30, and *N_w_* = 20 (*N_I_* and *N_w_* represent the numbers of IDT pairs and the multiple of the acoustic aperture to the wavelength, respectively) and with no SiO_2_ loading. Their electrode-overlap areas were kept equal, so changes in admittance arise primarily from boundary topology rather than from capacitance or active-area differences.

All layouts preserve a piston-like *A*_1_ maximum within the IDT overlap. Nevertheless, strong local deformation appears near IDT-to-busbar junctions. Straight, parallel edges allow the returned waves to remain coherent and form transverse standing waves, whereas curved or nonparallel boundaries distribute reflections across different phases and path lengths. The rectangle consequently produces the strongest spurious response (Figure 6a). Eye-shaped and cloud-shaped (Figure 6c,d) contours weaken several peaks, while the irregular pentagon offers the best practical compromise: unequal edge lengths and nonparallel sides disrupt a dominant round trip without imposing excessive curvature or fabrication burden. The improvement is therefore attributed to redistributed edge reflection rather than a different fundamental excitation mechanism.

**Figure 7:**
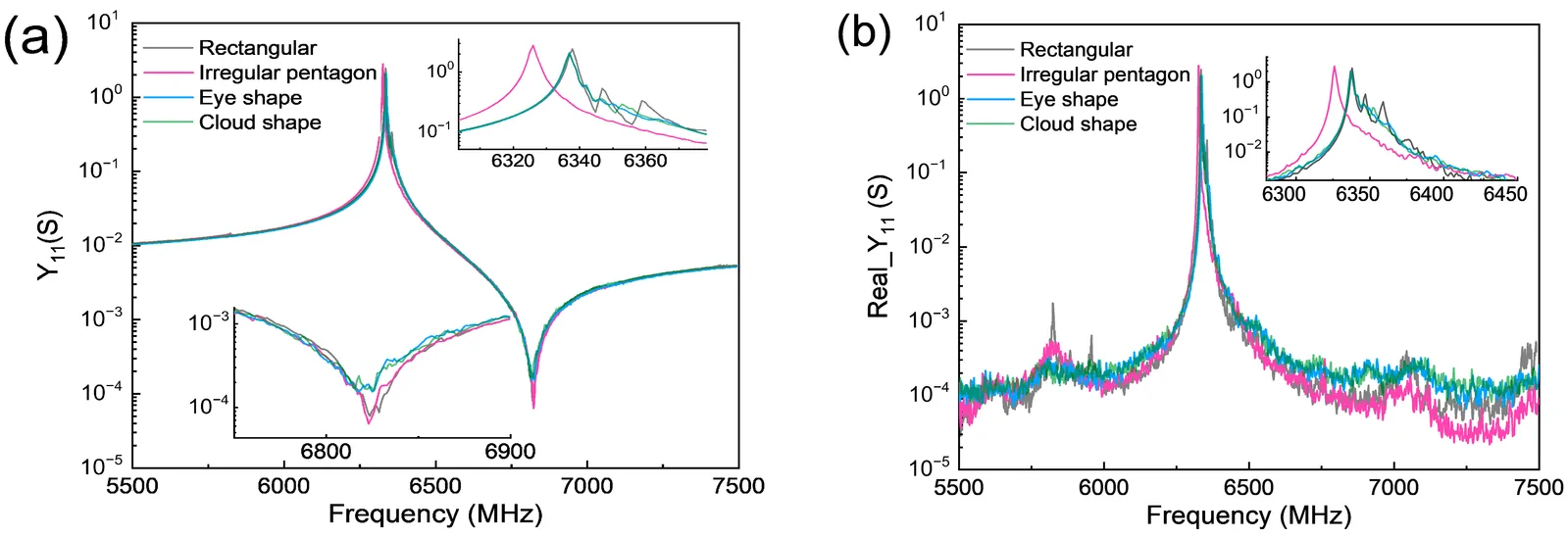
Electrical responses of LT-XBAR devices with rectangular, irregular pentagonal, eye-shaped, and cloud-shaped geometries. Comparison of (**a**) the input admittance *Y*_11_ and (**b**) the conductance *G* for the devices.

The electrical results in Figure 7 support this interpretation. The rectangular resonator shows clear parasitic features in input admittance and conductance near the main resonance. These features are less pronounced for the eye-shaped and cloud-shaped membranes and are strongly reduced for the irregular pentagon. Because the electrode-overlap area is unchanged, the smoother spectrum is consistent with the altered phase and direction of waves returning from the membrane edge. Table 1 summarizes the effective coupling values and the qualitative spurious-response comparison for these four geometries.

Compared with the rectangle, the irregular pentagon slightly lowers the resonance frequency and increases the effective coupling from 16.39% to 16.74%, a gain of 0.35 percentage points. Its real admittance above resonance is also lower over a broad interval. The combination of preserved coupling and a smoother response near the main mode motivates the selection of the irregular pentagonal membrane for the subsequent COM-based filter synthesis. The spurious-response comparison in Table 1 describes the spectral features visible in Figure 7; it does not assign a numerical suppression ratio.

The FEM results define a practical hierarchy. Orientation first establishes available coupling and velocity. LT thickness then sets the *A*_1_ mode frequency; wavelength and Al thickness tune capacitance, impedance, and secondary resonances. SiO_2_ loading provides the series–shunt offset. Finally, membrane contour, busbar junctions, aperture, and terminations are adjusted to suppress boundary modes. Following this order avoids mixing material, periodic-cell, and finite-device effects.

The unit cell provides reference velocity, transduction, reflection, capacitance, and loss trends for COM, whereas the complete model identifies finite-aperture and topology corrections. The FEM is therefore retained for physics requiring a field solution; repeated resonator and filter evaluations are transferred to the reduced model.

### 2.2. Fast Optimization Design Approach for XBAR Filters

A full-wave FEM is essential for identifying modes and analyzing boundaries, but repeated resonator retuning renders it too costly for filter optimization. Lumped BVD circuits are fast but neglect distributed wave interactions. The COM model offers a practical compromise: it retains propagation, reflection, transduction, capacitance, and loss in a compact network. After calibration against the preceding FEM results, it rapidly evaluates series and shunt resonators within the optimizer.

#### 2.2.1. COM Model for LT-XBAR

Coupling-of-modes theory links full-wave solutions to circuit-level admittance via a small set of coefficients that describe propagation, distributed reflection, electroacoustic conversion, and static capacitance. For periodic resonators, these local parameters enable closed-form transfer- or P-matrix formulations [33]. Conventional COM becomes less accurate for *shear-horizontal* (*SH*) waves near the fast shear bulk-wave velocity, where the penetration depth changes rapidly, the upper surface branch may delocalize, and radiation loss becomes frequency-dependent. Here, the same physical framework is retained for the 120° YX-LiTaO_3_ XBAR, with effective coefficients fitted continuously as a function of SiO_2_ thickness.

Let *R*(*x*) and *S*(*x*) denote the slowly varying forward and backward acoustic amplitudes, and let *V* and *I* denote the terminal voltage and current. A reciprocal uniform transducer satisfies:

(1) $$\left\{\begin{array}{l} \frac{\mathrm{dR}}{\mathrm{dx}}=-j\delta R-j\kappa S+j\alpha V\\ \frac{\mathrm{dS}}{\mathrm{dx}}=j\kappa^{*}R+j\delta S-j\alpha^{*}V\\ \frac{\mathrm{dI}}{\mathrm{dx}}=-4j\alpha^{*}R-4j\alpha S+j\omega CV \end{array}\right.$$

Here, *δ* is the detuning, *κ* is the distributed reflection, *α* is the transduction coefficient, and *C* is the capacitance per unit length. Applying the acoustic boundary conditions yields the P-matrix, whose *P*_33_ element equals the electrical admittance *Y*_11_ and can therefore be compared directly with the simulated complex admittance, and:

(2) $$P_{33}=P_{33}^{E}+P_{33}^{\mathrm{SC}}$$

In Equation (2), *P*_33_*^E^* describes currents related to the excited field, and the *P*_33_*^sc^* term can be used to fit the ripples on the admittance curve; in long IDT structures, the former term dominates. The detailed expression of *P*_33_ is given in Ref. [33]. Propagation and radiation losses are included through complex detuning, following Hashimoto’s general COM treatment [34,35].

In the two-parameter COM description of periodic gratings, *ε* controls reflection, and *η* characterizes surface localization (effective penetration depth). The wavenumber is:

(3) $$\theta_{p}=c\sqrt{\Delta^{2}-\frac{1}{4}{\left({\left|\varepsilon \right|}^{2}+\eta \sqrt{2{\left|\varepsilon \right|}^{2}-\eta^{2}-4\Delta }\right)}^{2}}$$

where *c* is a near-unity correction, and Δ is the normalized frequency detuning. The lower and upper stopband-edge frequencies are [33,36]:

(4) $$\left\{\begin{array}{l} \Delta_{-}=-\frac{{\left(\eta +\left|\varepsilon \right|\right)}^{2}}{2}\\ \Delta_{+}=-\frac{{\left(\eta -\left|\varepsilon \right|\right)}^{2}}{2}\;\mathrm{for}\;\eta >2\left|\varepsilon \right| \end{array}\right.$$

These relations capture both stopband edges and the loss of the upper surface-wave branch as its energy penetrates the bulk. Because *η* and *ε* can be estimated from the edge frequencies, they also provide stable initial values. Keeping *η* and *ε* separate is more physically meaningful than representing the *SH* response with an unconstrained empirical pole pair.

The two-parameter model and the extended surface-transverse-wave COM system were developed by Abbott and Hashimoto [37,38]. For the present XBAR geometry, *η*, *ε*, and *γ_p_* are fitted directly as dimensionless functions of SiO_2_ thickness. The dimensional coefficients used in COM are recovered using the lateral acoustic wavelength *λ* = 6 μm, yielding:

(5) $$\left\{\begin{array}{l} \eta_{s}=\frac{\eta }{\sqrt{\lambda }},\varepsilon_{s}=\frac{\varepsilon }{\sqrt{\lambda }}\\ \Delta_{v}=-\frac{\eta_{s}^{2}}{2},\eta_{B}=\frac{\eta_{s}}{2}+\varepsilon_{s}\\ \Delta_{B}=-\frac{\eta_{s}^{2}-2\varepsilon_{s}^{2}}{4}\\ v_{\Delta }=\frac{\eta_{B}}{\sqrt{\Delta_{B}-\Delta }+\eta_{B}}\\ \kappa =\varepsilon_{s}(\eta_{s}+\frac{\varepsilon_{s}}{2}),\kappa_{B}=-\frac{\varepsilon_{s}^{2}\eta_{s}}{\eta_{s}+2\varepsilon_{s}}\\ \theta_{u}=\Delta -\Delta_{v}+\left|\kappa_{B}\right|v_{\Delta }-j\frac{\gamma_{p}}{\lambda }\\ k=\kappa +\kappa_{B}v_{\Delta } \end{array}\right.$$

Here, Δ*_v_* sets the stopband center in the weak-interaction limit; *κ* denotes surface-wave coupling; and *κ_B_* and *ν*_Δ_ describe coupling to the counterpropagating shear bulk wave. The fitted *η*, *ε*, and *γ_p_* remain dimensionless, whereas *η_s_*, *ε_s_*, and *Γ_p_* have units of m^−1/2^, m^−1/2^, and m^−1^, respectively. This conversion preserves the closed-form COM structure while accounting for frequency-dependent localization and bulk-wave scattering omitted by the conventional constant-*κ* approximation.

The dimensionless loss parameter *γ_p_* enters the complex detuning via *Γ_p_ = γ_p_/λ*. The fitted electromechanical conversion coefficient is denoted by *α_p_* and multiplies the nominal transduction, *α_n_ = α_p_α*_0_. For a uniform section with *N_I_* periods and aperture factor *N_W_*, and within a specific frequency range, the acoustic and total admittances can be reformulated as:

(6) $$\left\{\begin{array}{l} Y_{\mathrm{ac}}=-\frac{4j\alpha_{n}^{2}}{\lambda }\frac{N_{W}N_{I}}{\theta_{u}-k}\\ Y=Y_{\mathrm{ac}}+j\omega C_{\mathrm{eff}}e^{-\rho (f_{\mathrm{GHz}}-4.75)} \end{array}\right.$$

To prevent the background capacitance from trading off freely with the motional branch, *C_eff_* is anchored to the low-activity region of the simulated spectrum:

(7) Ceff=median4.5–5.0 GHzIm(Y)2πfρ=0.163769 GHz−1

Only a shared smooth roll-off *ρ* is used, which reduces correlation among static capacitance, transduction, and the Plessky reflection parameters.

**Figure 8:**
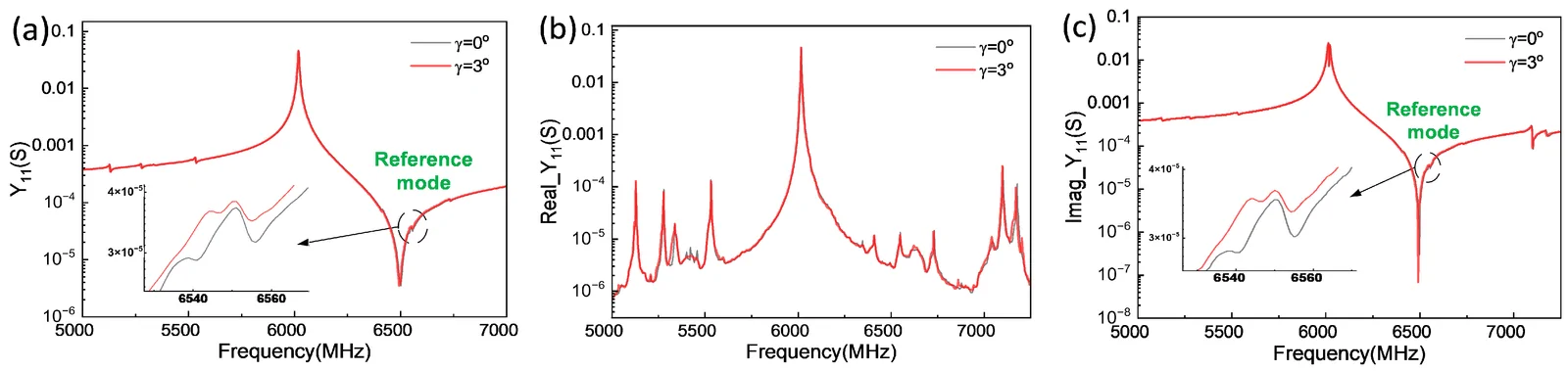
Simulated responses of Y-cut LiTaO_3_ XBARs at additional rotation angles of *γ* = 0° and 3°. The device dimensions are *N_W_* = 15, *λ* = 6 μm, and *t_SiO_*_2_ = 50 nm. The circled region indicates the reference mode. Subfigures show (**a**) the input admittance *Y*_11_, (**b**) its real component, and (**c**) its imaginary component.

For the COM model, establishing an accurate acoustic velocity reference is critical, as it directly affects COM parameter extraction and device-response prediction. In the modeling of leaky surface acoustic wave (LSAW) devices, for example, the surface skimming bulk acoustic wave (SSBAW) near the anti-resonance frequency *f_ar_* is commonly adopted as the reference point. For LT-XBAR device modeling, an additional rotation angle *γ* is introduced to identify this reference point. Specifically, by adding an additional 3°*γ* rotation to the Euler angles, the reference velocity *V_ref_* can be determined. Figure 8 presents the simulation results. A weak resonance mode appears near the *A*_1_ anti-resonance. Using the velocity associated with this mode as the reference provides an effective basis for constructing the COM model of XBAR devices with an SiO_2_ mass-loading layer. This choice improves parameter-extraction consistency and the reliability of model predictions. The FEM comparison in Figure 8 provides an angular-sensitivity check for *N_W_* = 15, *λ* = 6 μm, and *t*_sio2_ = 50 nm. Increasing *γ* from 0° to 3° shifts the principal resonance by about 2 MHz and the deep antiresonance by approximately 5–6 MHz, while the magnitude and complex-admittance responses remain nearly coincident elsewhere. Angular perturbation therefore has little effect on the main mode but is more visible near spectral zeros and parasitic features.

For a continuous thickness model, define the parameter *u =* (*t_SiO_*_2_ − 40)/30, where *t_SiO_*_2_ is the SiO_2_ thickness in nanometers. Reference velocity, capacitance, transduction, loss, penetration, and reflection are described by the joint laws:

(8) $$\left\{\begin{array}{l} V_{\mathrm{ref}}=4300\exp(-0.036254-0.012u-0.0012287u^{2})\left[m/s\right]\\ C_{\mathrm{eff}}=\left(1.08989+0.0424419u+0.00597045u^{2}\right)\times 10^{-14}\left[F\right]\\ \alpha =\exp(-0.163628-0.00321899u-0.00248902u^{2})\\ \gamma_{p}=\exp(-5.63447+0.306899u+0.286786u^{2})\\ \eta =\exp(0.501467+0.0542598u-0.0318654u^{2})\\ \varepsilon =\exp(-1.23211+0.229928u+0.216972u^{2}) \end{array}\right.$$

Admittance spectra across all SiO_2_ thicknesses were fitted jointly. The objective combines clipped magnitude residuals with normalized real- and imaginary-admittance residuals, using Gaussian weights near resonance and antiresonance. Exponential parameterization keeps each scale positive, while a weak monotonic constraint on reference velocity (*V_ref_*) limits unphysical trade-offs between phase velocity and *ε* or *η*.

**Figure 9:**
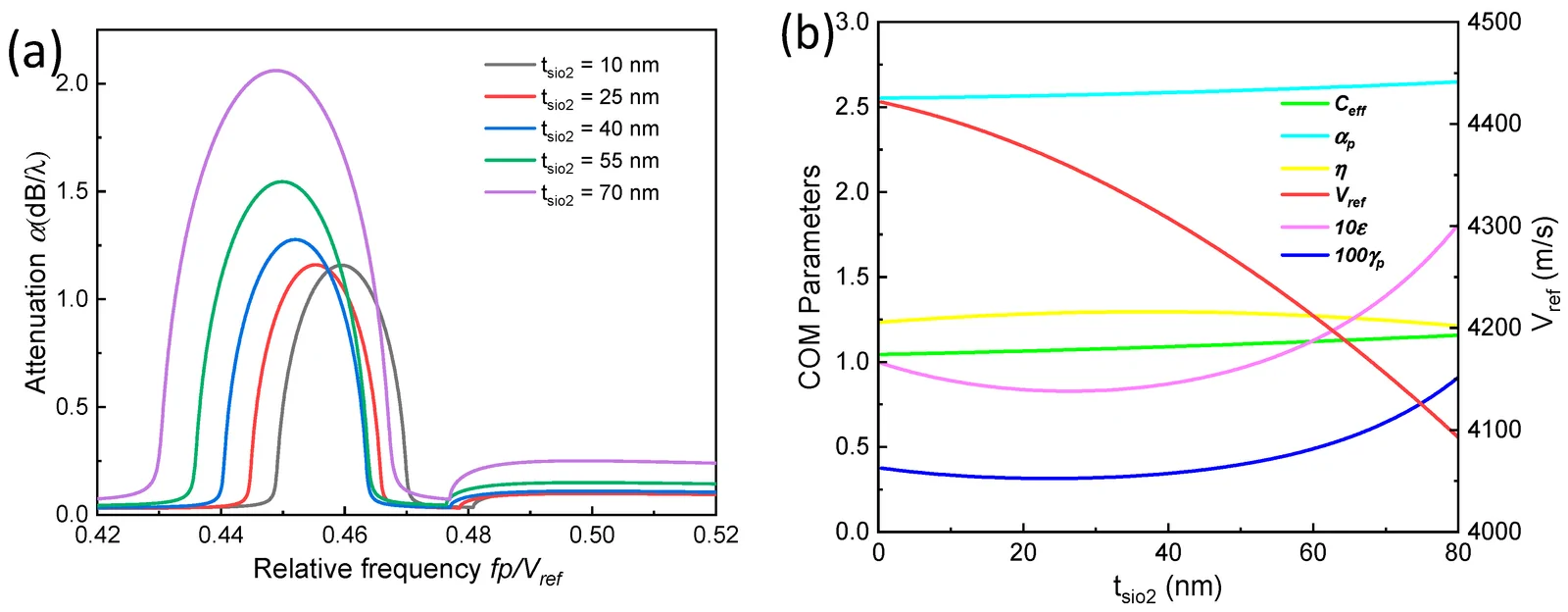
(**a**) Thickness-dependent attenuation calculated from the fitted Plessky-COM propagation constant; (**b**) fitted COM parameters and the reference velocity as functions of SiO_2_ thickness.

As shown in Figure 9a, increasing SiO_2_ thickness strengthens the main attenuation peak and shifts the stopband toward lower normalized frequency. The calculated maximum increases from approximately 1.15 dB per wavelength at 10 nm to more than 2.0 dB per wavelength at 70 nm. Thus, the overlayer affects both distributed reflection and spectral position, not merely static capacitance. Figure 9b summarizes the fitted trends. As *t_SiO_*_2_ increases, *V_ref_* decreases from about 4403 to 4092 m/s, while *C_eff_* rises from 1.05 × 10^−14^ to 1.14 × 10^−14^ F. The *η* scale gradually saturates, whereas *ε* and *γ_p_* increase more strongly at larger thicknesses, consistent with changes in *SH*-wave localization, reflection, and damping.

The joint fit yields a mean magnitude-domain RMSE (Root Mean Square Error) of 0.997 dB. For most thicknesses, resonance and antiresonance positions agree within 0–1 MHz; the fitted quantities should therefore be viewed as effective parameters for this geometry and frequency band, rather than as independent material constants. Over 0–80 nm, the model provides physically traceable and efficient interpolation for XBAR design sweeps and circuit-level *Y*_11_ prediction.

**Figure 10:**
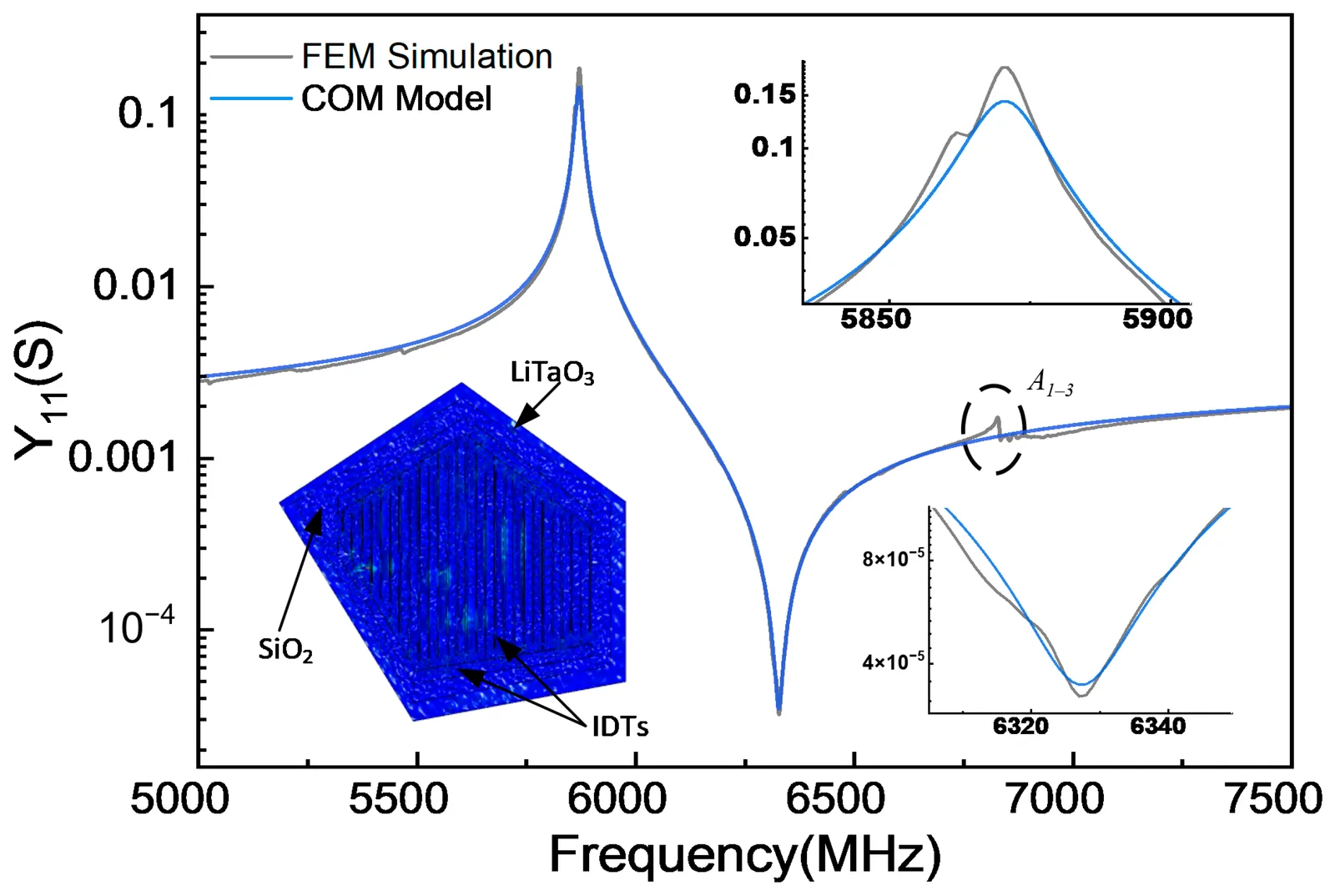
Simulated electrical responses of irregular pentagonal LT-XBAR devices with a 70 nm SiO_2_ loading layer. Insets show enlarged resonance and antiresonance regions.

Figure 10 validates the extracted COM model against a full-wave FEM model of a shunt resonator with a 70 nm SiO_2_ layer. For the irregular pentagonal LT-XBAR, which suppresses spurious modes near resonance (*f_r_*), the COM result from Equations (6)–(8) closely reproduces the overall *Y*_11_ spectrum and the enlarged resonance and antiresonance regions without repeating a full-field solution for every candidate. Small local differences likely arise from higher-order field effects (e.g., the horizontal *A*_1–3_ harmonic close to the right of anti-resonance [7]).

#### 2.2.2. Artificial Bee Colony (ABC) Optimization

The artificial bee colony (ABC) algorithm serves as the outer optimizer because ladder-filter design involves both discrete and continuous variables and yields a non-smooth objective function. A candidate filter with *N_r_* resonators is represented by the design vector *x*, which contains the IDT finger-pair number *N*_*I*,*r*_, normalized aperture *N*_*W*,*r*_, and wavelength *λ_r_* of each resonator (Table 2):

(9) $$x=\left[N_{I,1},N_{W,1},\lambda_{1},\right.\cdots ,N_{I,N_{r}},N_{W,N_{r}},{\left.\lambda_{N_{r}}\right]}^{T}$$

Here, *r* = 1, ⋯, *N_r_*; *N*_*I*,*r*_ and *N*_*W*,*r*_ are integer variables, whereas *λ_r_* varies continuously within the allowable lithographic range. Passband and stopband extrema are obtained from the cascaded filter response, so gradient information is unreliable near discrete transitions. ABC therefore searches the resonator parameters without derivatives, while the calibrated COM model evaluates each candidate.

Although each variable has a primary physical role, their effects are coupled. Wavelength shifts resonance via the phase-velocity-to-period relation, finger-pair number changes transduction length, conductance, and static capacitance, and aperture controls active area, impedance, and loss. Because a wavelength-induced pole shift can be partially compensated by finger count or aperture, a population-based, filter-level search is preferable to coordinate-wise tuning.

The variables are subject to the following physical bounds:

(10) $$x_{j}^{\min}\leq x_{j}\leq x_{j}^{\max},j=1\cdots D$$

(11) $$x=x_{j}^{\min}+\mathrm{rand}\left(0,1\right)\cdot \left(x_{j}^{\max}-x_{j}^{\min}\right),i=1\cdots \mathrm{SN},j=1\cdots D$$

Equation (10) specifies the admissible design space, and Equation (11) initializes *SN* (solution number) food sources. Each source represents a filter. The bounds keep all candidates within the calibrated COM domain and lithographic limits before evaluating their network responses.

In the employed-bee phase, each food source *x_i_* is updated by perturbing one of its design variables relative to a randomly selected neighboring source *x_k_*. For the *j*-th coordinate, the trial value is computed as:

*V_ij_* = *x_ij_* + *φ_ij_*·(*x_ij_* − *x_kj_*), *k* ≠ *i* (12)

Here, *φ_ij_* is a uniformly distributed random coefficient, and *k* denotes a randomly selected source different from *i*. The resulting trial vector is decoded into the physical filter variables; integer-valued entries are rounded, and any constraint violations are repaired before the candidate response and objective cost are evaluated. Greedy selection retains the trial solution only if its cost does not exceed that of its parent.

In the onlooker-bee phase, sources are selected based on their normalized fitness values, so that candidates with lower objective costs have a higher probability of further exploration. For a non-negative minimization cost *J_i_*, the selection probability for source *i* is defined as:

(13) $$P_{i}=\frac{\mathrm{fit}}{\sum_{n}\mathrm{fi}t_{n}}$$

For each candidate, COM calculates the resonators’ admittances, which are then cascaded using ABCD matrices to obtain the filter response. Passband loss and ripple, stopband leakage, band-edge errors, and feasibility violations are combined into a weighted cost. Normalization by suitable tolerances prevents any term from dominating due to its numerical scale.

Every candidate is evaluated on the same dense frequency grid. The simplified COM model is used for optimization, and selected solutions are verified with the complete COM model and FEM-derived admittance cascades. Metrics are compared over identical windows to reduce artifacts from model simplification or inadequate sampling. The objective is:

(14) $$\begin{array}{ll} \mathrm{Cost}\left(x\right)= & \omega_{\mathrm{IL}}\cdot \max\left(0,\mathrm{IL}-IL_{\mathrm{tar}}\right)+\omega_{\mathrm{Rip}}\cdot \max\left(0,\right.\mathrm{Rip}-\left.\mathrm{Ri}p_{\mathrm{tar}}\right)+\omega_{\mathrm{Rej}}\cdot \max(0,{\mathrm{Rej}}_{\mathrm{tar}}-\mathrm{Rej})\\ \; & \;+\omega_{\mathrm{BW}}\cdot \max\left(0,BW_{\mathrm{tar}}-\mathrm{BW}\right)+\omega_{\mathrm{fc}}\cdot \frac{\left|f_{c}\left(x\right)-\left.f_{0}\right|\right.}{\Delta f} \end{array}$$

Equation (14) defines a weighted hinge-penalty objective. Its terms represent worst-case insertion loss, passband ripple, stopband rejection, bandwidth, and center-frequency deviation. A penalty is active only when its specification is violated, and the weights determine relative importance. Minimizing this cost therefore balances passband, stopband, bandwidth, center-frequency, and manufacturability requirements rather than optimizing a single resonator metric.

**Figure 11:**
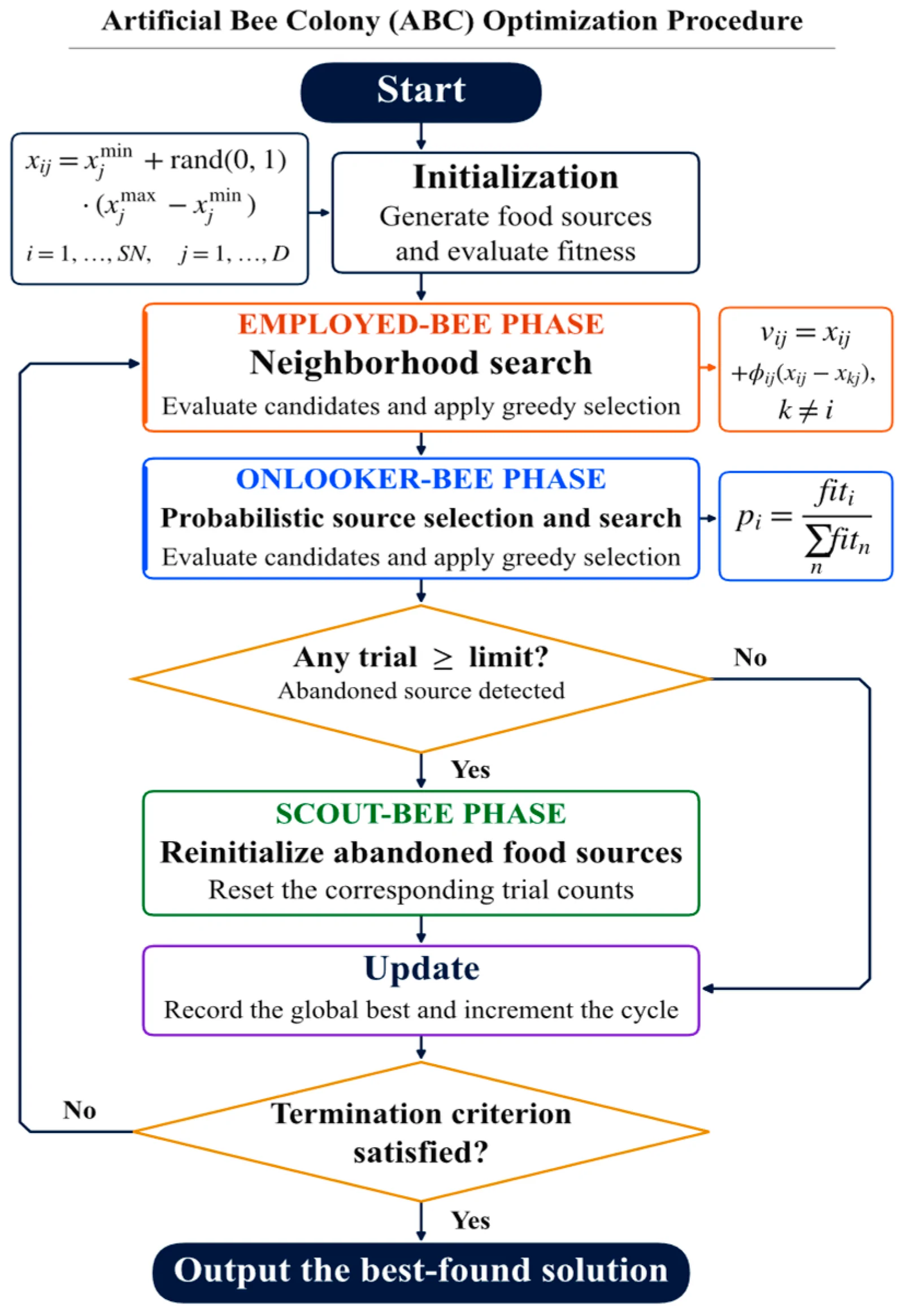
Practical optimization sequence for the ABC algorithm. Orange, blue, and green outlines identify the employed-, onlooker-, and scout-bee phases, respectively; purple identifies the global-best and cycle update, and gold diamonds indicate decision steps.

Figure 11 summarizes the implementation. Each source encodes a complete LT-XBAR ladder filter. Employed and onlooker bees update continuous coordinates. *N_I_* and *N_W_* are decoded as discrete values, and each trial is repaired before evaluation and greedy replacement. The COM/ABCD cascade serves as the fitness evaluator. Scout replacement, partial restart, elite retention, and termination make the mixed-variable search reproducible and auditable.

## 3. Results

### 3.1. Bandpass Filter Synthesis Case Study

The selected design is parameterized in physical wavelengths (micrometers). Series resonators remain uncovered, whereas all shunt resonators carry a common 70 nm SiO_2_ layer. For the LT-XBAR-based bandpass filter design and optimization, the critical parameters *N_I_* and *N_W_* are optimized separately for each element, allowing for local adjustment of conductance and static capacitance. The reported acoustic-only comparison does not use a matching inductor to increase out-of-band rejection.

To examine wavelength freedom on its own, we consider two otherwise identical five-stage filters synthesis problems. Case I fixes the wavelength of all ten resonators at 6 μm; parameters *N_I_* and *N_W_* can still vary by stage. Case II then allows the acoustic wavelength *λ_r_* of each series and shunt resonator to vary independently in addition to those two variables. Both cases retain the same material stack and series/shunt topology, with 70 nm SiO_2_ loading on the shunt arms. This sequence follows a practical design progression: first establish the performance attainable with a single 6 μm IDT period, and then relax the wavelength constraint to assess the benefit of a stage-dependent IDT period. The topology, material stack, boundary conditions, frequency grid, and objective weights remain identical.

For each stage of Case I, *N*_*I*,*r*_ and *N*_*W*,*r*_ are reoptimized to achieve the required impedance transformation across the entire ladder network. The resulting stage parameters may therefore differ, especially near the ladder terminations. However, extreme integer values or very small apertures can increase sensitivity to electrode resistance, edge placement, and transverse modes. A manufacturing-aware refinement should therefore impose process-derived bounds and penalize sensitivity to pitch, aperture, film thickness, and material constants.

**Figure 12:**
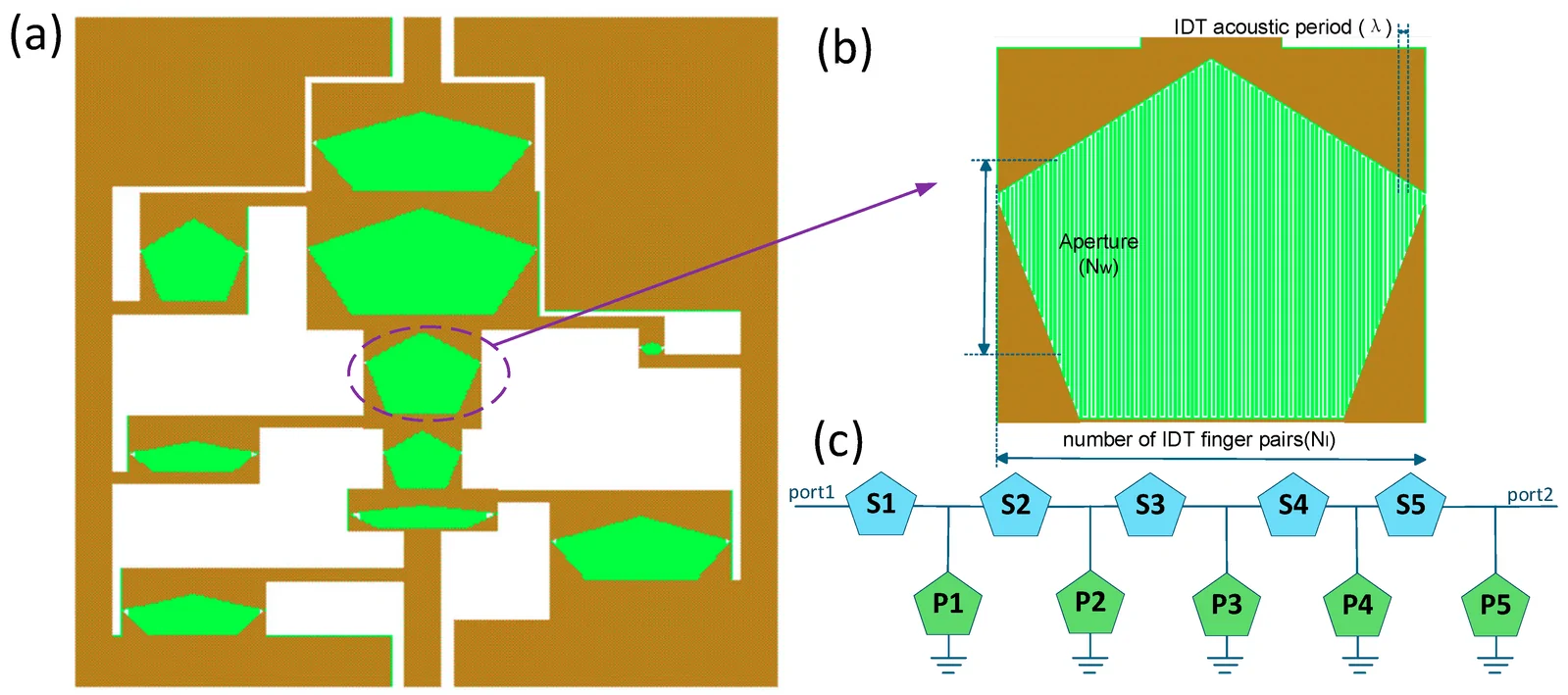
Five-stage LT-XBAR ladder filter: (**a**) physical layout with irregular pentagonal resonators; (**b**) enlarged IDT geometry showing the pentagonal arrangement of IDTs; (**c**) circuit topology with five series resonators (S1–S5) and five grounded shunt resonators (P1–P5).

The optimized Case I parameters are listed in Table 3, and Figure 12 shows the physical layout, IDT geometry, and ladder topology. To ensure a fair comparison with the rectangular reference design, each pentagonal resonator is assigned the same active IDT area as its corresponding rectangular resonator, i.e., *A_r_* = *N_I_*_,*r*_∙*N_W_*_,*r*_∙*λ_r_*^2^.

For circuit-level verification based on FEM-derived resonator responses, the complex admittances of all ten resonators are interpolated onto a common frequency grid. For each series resonator, the admittance *Y* is converted to the impedance *Z* = 1/*Y* and represented by the ABCD matrix $\left[\begin{array}{cc} 1 & Z\\ 0 & 1 \end{array}\right]$. Each shunt resonator is represented by $\left[\begin{array}{cc} 1 & 0\\ Y & 1 \end{array}\right]$. The matrices are multiplied in physical ladder order to obtain the overall ABCD matrix, from which the transmission coefficient *S*_21_ is calculated for equal source and load impedances, as follows:

(15) $$S_{21}=\frac{2}{A+\frac{B}{Z_{0}}+C\cdot Z_{0}+D}$$

**Figure 13:**
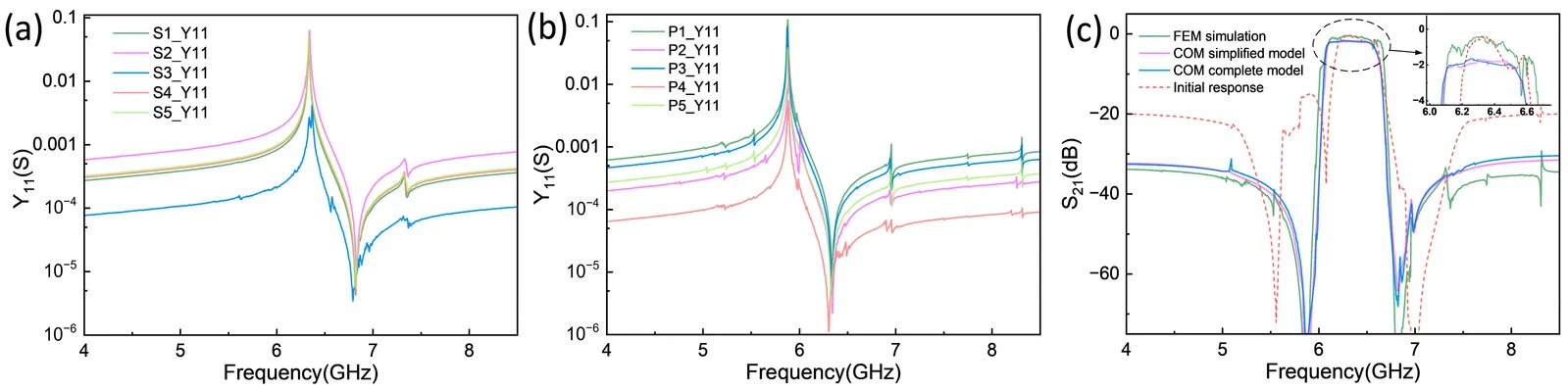
Comparison of the directly cascaded FEM ladder, the COM simplified model, and the COM complete model for Case I, with *λ* = 6 μm for all resonators. The admittance spectra of (**a**) the series and (**b**) shunt resonators; (**c**) comparison of the *S*_21_ responses with different models.

Figure 13 and Table 4 compare the Case I responses from the FEM-derived admittance cascade, the simplified COM model, and the complete COM model. All three approaches predict the same principal passband near 6.3 GHz, indicating that the COM models capture the dominant filter behavior. Using the FEM-derived cascade as the reference, the peak *S*_21_, passband ripple, and stopband rejection are −0.40 dB, 1.10 dB, and 29.28 dB, respectively. The simplified and complete COM models yield peak *S*_21_ values of −1.67 and −1.65 dB, respectively, approximately 1.27 and 1.25 dB lower than the reference value. Discrepancies are observed in the out-of-band rejection within the left high-frequency band, with the full FEM model yielding higher rejection, which is attributed to the omission of the higher-order lateral harmonics (*A*_1–3_*, A*_1–5_) that occur above *f_ar_* for both series (~7.4 GHz) and shunt (~7 GHz) resonators, an effect not included in the COM formulation. Although quantitative differences remain in the peak transmission, ripple, and stopband response, the COM models reproduce the passband location and overall response shape. More importantly, the COM/ABCD implementation evaluates each candidate using compact circuit-level calculations, avoiding repeated full three-dimensional FEM simulations during the ABC optimization loop. It therefore provides an efficient surrogate model for filter synthesis, while the FEM-derived cascade is retained to validate the selected design. The −3 dB bandwidth of Case I is approximately 596 MHz.

**Figure 14:**
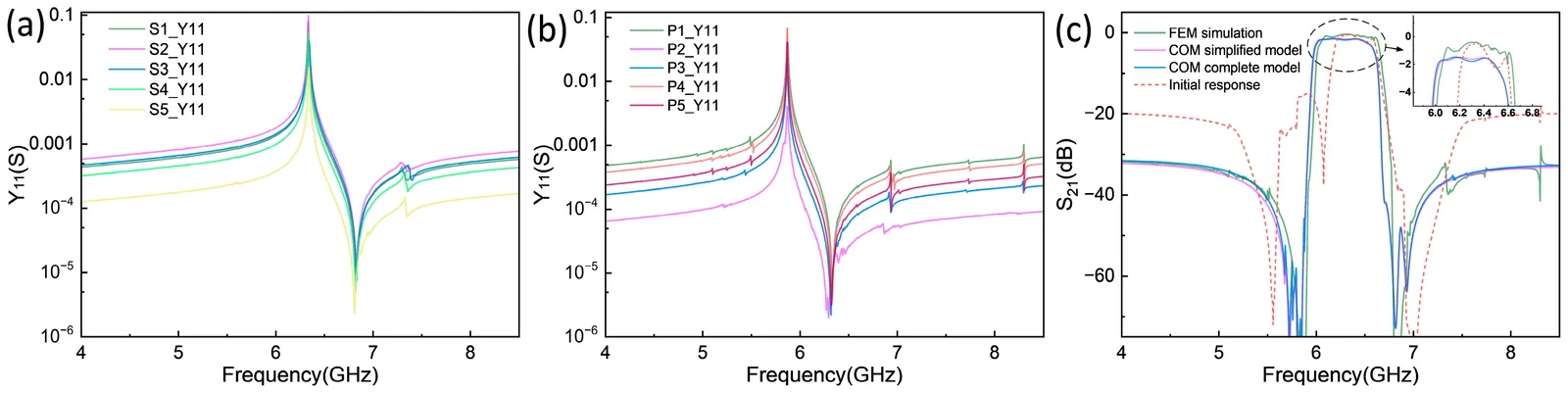
Resonator admittances and *S*_21_ responses for Case II, in which the wavelength is optimized independently for each resonator. The admittance spectra of (**a**) the series and (**b**) shunt resonators; (**c**) comparison of the *S*_21_ responses with different models.

In Case I, we considered only the effects of *N_I_* and *N_W_* on the filter, which were critical to optimizing the LT-XBAR filter’s primary characteristics. However, to further enhance the filter performance, a viable design strategy is to introduce additional variables, such as the wavelength of the XBAR resonators and the thickness of the mass-loading layer on the shunt arms. Given practical fabrication constraints, adjusting the mass-loading thickness individually for each shunt resonator would substantially increase process difficulty and cost. Therefore, we focus on tuning each resonator’s wavelength. As shown in the finite-element simulation results in Figure 4a, although the LT-XBAR resonator is not particularly sensitive to wavelength changes, proper tuning of individual resonant wavelengths can suppress overlap of spurious modes between the series and shunt branches, helping flatten the in-band response. In addition, judicious wavelength adjustment can widen the device bandwidth, a common practice in commercial ladder-type SAW filters, where each resonator typically operates at a distinct wavelength. Consequently, we further investigate how resonator wavelength variation affects filter performance. By introducing *λ_r_* as an extra variable and employing the combined COM/ABC algorithm, we designed a new fifth-order LT-XBAR bandpass filter; its key structural parameters are summarized in Table 5. Figure 14c presents the *S*_21_ curve of the further optimized filter, and Table 6 summarizes the new filter’s response metrics.

Table 5 presents the performance-oriented design: its optimized wavelengths span 5.8967–6.2000 μm. Adding wavelength as a stage-specific variable allows the optimizer to coordinate acoustic pitch, finger-pair number, and normalized aperture, providing more direct control of the pole-zero distribution. The comparison between Table 4 and Table 6 therefore treats Case I as the manufacturing-oriented baseline and Case II as the wavelength-optimized design.

The optimized Case II yields an approximately 606 MHz −3 dB bandwidth, compared with about 596 MHz for Case I. As summarized in Table 4 and Table 6, both COM models predict a 0.19 dB improvement in peak *S*_21_: the simplified model improves from −1.67 to −1.48 dB and the complete model from −1.65 to −1.46 dB. The corresponding ripple decreases from 0.53 to 0.28 dB and from 0.46 to 0.30 dB, respectively. The FEM-derived cascade shows the same in-band trend, with the peak *S*_21_ improving from −0.40 to −0.38 dB and the ripple decreasing from 1.10 to 0.70 dB. These consistent improvements indicate that independent wavelength tuning is effective for passband optimization.

### 3.2. Repeatability of ABC Optimization

To assess the repeatability of the stochastic ABC search, ten independent runs were performed for each of Case I and Case II. Within each case, the target frequency bands, objective function and weights, parameter bounds, population size, and iteration count were held fixed; only the random initialization was varied. The FEM-based worst-case passband insertion loss (IL), peak-to-peak passband ripple, and stopband rejection were recorded for each run using consistent evaluation windows and metric definitions. Table 7 summarizes the mean, sample standard deviation, and observed range of each metric; the individual results are listed in Appendix A.

Here, IL denotes the largest insertion loss within the prescribed passband and is expressed as a positive loss. It therefore differs from the minimum insertion loss inferred from the peak S_21_ values in Table 4 and Table 6. Those tables compare selected individual designs across models, whereas Table 7 characterizes the distribution of the additional independent optimization runs.

In these ten-run ensembles, Case II has lower mean worst-case insertion loss and passband ripple than Case I: 1.904 versus 2.283 dB and 0.368 versus 0.449 dB, respectively. The corresponding reductions in the means are 0.379 and 0.081 dB. Case II also shows smaller sample standard deviations for all three metrics: 0.111 versus 0.221 dB for IL, 0.063 versus 0.075 dB for ripple, and 0.406 versus 1.560 dB for rejection. Its mean rejection is slightly lower, at 41.371 versus 41.758 dB, so the passband improvement is accompanied by a small reduction in mean rejection.

These results quantify the observed variability of the optimization outcomes and support more consistent performance metrics for Case II under the tested settings. The conclusion is limited to the ten runs per case and does not imply identical optimized geometries, guaranteed convergence to a global optimum, or robustness to fabrication variations.

## 4. Discussion

### 4.1. Computational Cost

The FEM-COM-ABC workflow separates full-field model calibration from repeated filter evaluations. COMSOL Multiphysics 6.4 was used for the full-field FEM calculations, and MATLAB R2025a was used for the COM/ABCD evaluator and ABC search. Both calculations were CPU-based. The reduced-order evaluator cascades ten resonators in a five-stage ladder over 4.0–8.5 GHz. Table 8 summarizes the measured cost of the two computational stages.

The recorded full-field frequency sweep required more than 200 h and approximately 16.3 GB of memory. The ABC core search with 40 food sources and 5000 cycles required approximately 2.9 h and 1.91 GB for the MATLAB process. Its 400,040 objective evaluations comprise 40 initial evaluations and 400,000 employed/onlooker trials, before additional scout evaluations. For a fixed number of retained modes, each COM/ABCD response requires work proportional to the number of resonators and frequency samples; the repeated evaluation cost also grows approximately linearly with the number of food sources and cycles. The computational benefit is the removal of repeated full-field solves from the search. The reported times do not constitute a per-candidate speedup or the total design turnaround time.

### 4.2. Sensitivity to Fabrication Parameters

The resonator sweeps in Figure 4, Figure 5 and Figure 8 examine electrode geometry, dielectric-film thickness, and crystal orientation. Reducing the wavelength from 6.4 to 5.8 μm produces a small upward shift of the main resonance (Figure 4a), whereas increasing the Al thickness from 20 to 120 nm changes the resonance frequency by less than 30 MHz (Figure 4b). These results indicate modest main-resonance sensitivity over the investigated ranges, although coupling, impedance, and spurious features may respond differently. For the configuration in Figure 8, with a normalized aperture of 15, a wavelength of 6 μm, and 50 nm SiO_2_ loading, an additional rotation of *γ* from 0° to 3° shifts the principal resonance by approximately 2 MHz and the antiresonance by approximately 5–6 MHz. The angular change is therefore more visible near the antiresonance than near the main resonance.

**Figure 15:**
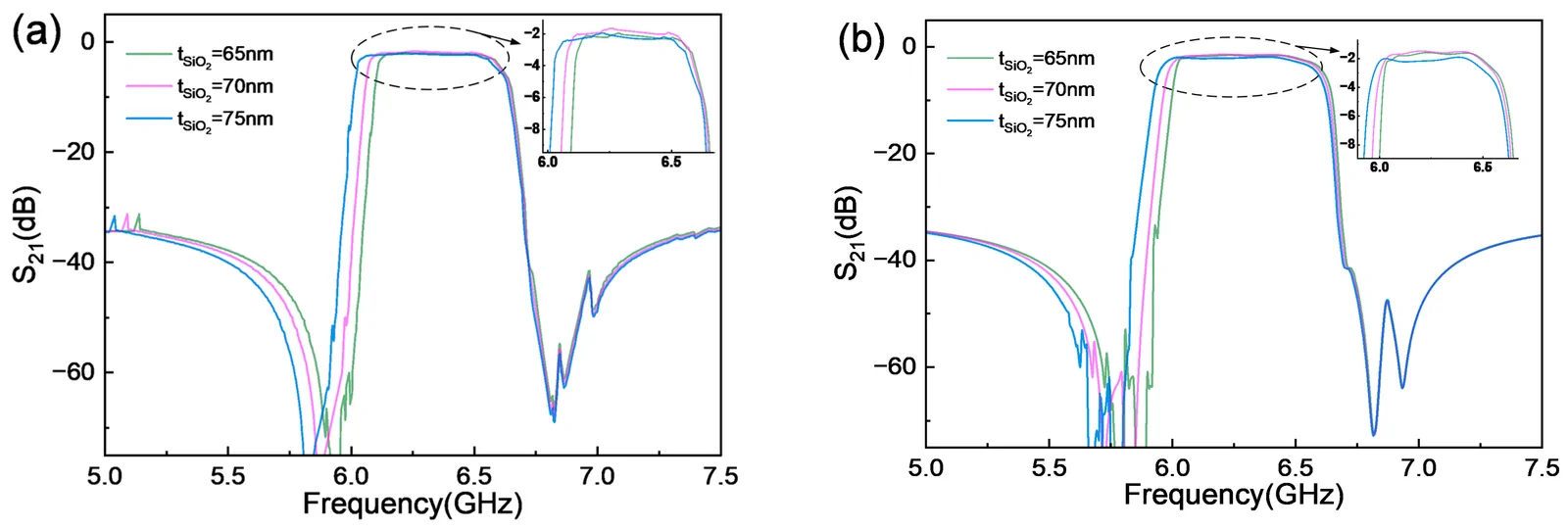
Main-mode S_21_ responses for separately optimized filters with shunt SiO_2_ thicknesses of 65, 70, and 75 nm: (**a**) Case I, with a common wavelength of 6 μm; (**b**) Case II, with independently optimized resonator wavelengths. Series resonators are uncoated. Insets show enlarged passband responses.

SiO_2_ loading directly sets the series–shunt frequency separation: increasing its thickness lowers the shunt resonance in Figure 5. Figure 15 extends this comparison to filter responses at shunt thicknesses of 65, 70, and 75 nm, corresponding to ±5 nm (approximately ±7.1%) around the nominal 70 nm value. The series resonators remain uncoated. At each thickness, the filter was optimized separately within the corresponding Case I or Case II design space. Case I retains a common 6 μm wavelength, whereas Case II allows individual resonator wavelengths.

Figure 15 shows that the separately optimized filters retain passbands near 6.3 GHz at all three shunt SiO_2_ thicknesses. In Case I, the 75 nm design has a lower upper passband edge than the 65 and 70 nm designs; in Case II, the 70 nm design extends to a higher upper passband edge than the other two designs. The enlarged views also reveal differences in passband transmission and ripple. These results demonstrate that optimization can recover similar passband locations at the tested coating thicknesses, while the band edges and out-of-band responses vary among the selected designs. Because each thickness was optimized separately, the differences also reflect changes in design parameters and may include search variability.

Together with the resonator-level sweeps, this comparison provides a limited numerical assessment of the effects of selected fabrication-related parameters on the modeled response. Sensitivity to LiTaO_3_ thickness and electrode finger width was not evaluated, as these dimensions were fixed at 300 nm and 500 nm, respectively. The conclusions are consequently limited to the parameters and ranges investigated here.

### 4.3. Model Scope and Limitations

The proposed 120° YX-LiTaO_3_ resonators and filters have been evaluated numerically. Experimental validation remains pending owing to limited access to suitable thin-film substrates with the required crystallographic orientation. Cascading individual FEM-derived resonator admittances omits inter-resonator coupling, interconnects, and packaging, so the predicted low minimum insertion loss remains a model-based result. The effective COM coefficients apply to the investigated geometry, frequency band, and material stack. Transfer to other cuts or geometries requires recalibration and validation. Higher-order lateral harmonics and residual scattering are not fully represented, which contributes to differences in transmission, ripple, and rejection despite close agreement in the principal resonance frequencies. Temperature dependence, power handling, and statistical fabrication yield have not been quantified.

## 5. Conclusions

This work presents an integrated FEM–COM–ABC framework for the numerical design of 120 °YX-LiTaO_3_ XBAR resonators and ladder filters. FEM analysis clarifies the influence of crystal orientation, electrode geometry, and SiO_2_ loading on the resonant response and guides the selection of an irregular pentagonal membrane, which suppresses spurious features near the main resonance and increases effective coupling from 16.39% to 16.74% relative to the rectangular design. The FEM-calibrated COM model achieves a mean magnitude-domain RMSE of 0.997 dB, with resonance and antiresonance frequency errors typically within 1 MHz. Coupling this model to ABC enables mixed-variable optimization of finger-pair number, aperture, and resonator wavelength without repeated full-field simulations. For five-stage filters operating near 6.3 GHz, independently optimized wavelengths in Case II increase the −3 dB bandwidth from approximately 596 to 606 MHz and improve COM peak transmission by 0.19 dB relative to the common 6 μm wavelength baseline. The FEM-derived admittance cascade also shows a ripple reduction from 1.10 to 0.70 dB, supporting the predicted improvement in nominal passband flatness. Ten independent runs per case yield lower mean worst-case insertion loss and ripple for Case II, together with smaller observed standard deviations in all three reported metrics, although its mean rejection is slightly lower. Separately optimized designs with 65–75 nm shunt SiO_2_ loading retain similar passband locations while exhibiting differences in band edges and out-of-band responses. These comparisons illustrate the framework’s ability to accommodate different coating thicknesses through design optimization. Future work should quantify fixed-design sensitivity to fabrication variations and experimentally validate the predicted filter performance.

## Figures and Tables

**Table 1:** Comparison of effective coupling and spurious responses for the four membrane geometries in Figure 7.

Membrane Geometry	Effective Coupling (%)	Spurious Response Near the Main Resonance (Qualitative)
Rectangular	16.39	Pronounced parasitic peaks
Irregular pentagonal	16.74	Strongly reduced parasitic features
Eye-shaped	16.25	Less pronounced spurious peaks; residual features remain
Cloud-shaped	16.48	Less pronounced spurious peaks; residual features remain

Note: All geometries use *λ* = 5.8 μm, *N_I_* = 30, *N_W_* = 20, no SiO_2_ loading, and the same active IDT overlap area. Spurious-response descriptions are qualitative assessments of Figure 7 near the main resonance.

**Table 2:** The notation used in the ABC algorithm.

Symbol	Definition
*IL*	Worst-case insertion loss within the prescribed passband (maximum loss adopted)
*Rip*	Peak-to-peak ripple within the passband
*Rej*	Maximum stopped rejection (a positive value denotes rejection capability)
*BW*	−3 dB bandwidth
*f_c_*(*x*)	Actual center frequency of the filter
*f*_0_	Target center frequency
Δ*f*	Frequency-normalization factor (typically set to *BW_tar_*)
*IL_tar_*,*Rip_tar_*	Corresponding design target values
*BW_tar_*	Target bandwidth
*ω_IL_*, *ω_Rip_*, *ω_Rej_*, *ω_BW_*, *ω_fc_*	Weighting coefficients for the individual terms

**Table 3:** Five-stage parameter set for Case I with a common resonator wavelength.

Unit	N_I_	N_W_	λ_r_ (μm)	SiO_2_ (nm)
S_1_	70	17	6.0	0
P_1_	50	36	6.0	70
S_2_	136	36	6.0	0
P_2_	11	12	6.0	70
S_3_	100	5	6.0	0
P_3_	68	27	6.0	70
S_4_	84	19	6.0	0
P_4_	25	4	6.0	70
S_5_	54	20	6.0	0
P_5_	87	16	6.0	70

**Table 4:** Response metrics for Case I with a common resonator wavelength.

Response	Peak S_21_	Ripple	Rejection
FEM simulation	−0.40 dB	1.10 dB	29.28 dB
Com simplified model	−1.67 dB	0.53 dB	31.55 dB
Com complete model	−1.65 dB	0.46 dB	30.43 dB

**Table 5:** Five-stage parameter set for Case II with independently optimized resonator wavelength.

Unit	N_I_	N_W_	λ_r_ (μm)	SiO_2_ (nm)
S_1_	103	27	6.1190	0
P_1_	50	28	6.0595	70
S_2_	107	36	6.1361	0
P_2_	11	4	6.2000	70
S_3_	56	29	5.8967	0
P_3_	60	10	6.1489	70
S_4_	37	20	6.0103	0
P_4_	85	22	6.0220	70
S_5_	70	8	5.9959	0
P_5_	67	14	6.0522	70

**Table 6:** Response metrics for Case II with independently optimized resonator wavelength.

Response	Peak S_21_	Ripple	Rejection
FEM simulation	−0.38 dB	0.70 dB	31.94 dB
Com simplified model	−1.48 dB	0.28 dB	32.26 dB
Com complete model	−1.46 dB	0.30 dB	31.54 dB

**Table 7:** Repeatability of FEM-based filter metrics over ten independent ABC runs per case.

Metric (dB)	Case I Mean ± SD	Case I Range	Case II Mean ± SD	Case II Range
Worst-case IL	2.283 ± 0.221	1.93–2.56	1.904 ± 0.111	1.76–2.07
Passband ripple	0.449 ± 0.075	0.33–0.54	0.368 ± 0.063	0.30–0.50
Stopband rejection	41.758 ± 1.560	39.39–44.59	41.371 ± 0.406	40.96–42.30

Note: Each case comprises *n* = 10 independent runs. SD is the sample standard deviation, calculated with denominator *n* − 1 = 9; ranges are the observed minimum and maximum. Statistics were computed from run-level metrics recorded to 0.01 dB. Lower IL and ripple are preferable, whereas higher rejection is preferable.

**Table 8:** Computational cost of the full-field FEM and reduced-order ABC stages.

Metric	Direct Full-Field FEM	FEM-COM-ABC	Basis
Execution engine	COMSOL Multiphysics, full-field FEM	MATLAB R2025a, COM/ABCD + ABC	CPU-only
Computation scope	One full 4.0–8.5 GHz frequency sweep	Complete ABC core loop: 40 sources, 5000 cycles	Different workflow stages
Reported computation time	>200 h	~2.9 h	FEM sweep vs. ABC core loop
Peak host memory	~16.3 GB	~1.91 GB (MATLAB process)	~88% lower

Note: The ABC timing covers initialization and employed/onlooker updates after model calibration. Calibration, scout replacements, interface overhead, and selected-design FEM validation are excluded; the two columns therefore describe different workflow stages.

## Data Availability

The data that support the findings of this study are available from the corresponding authors upon reasonable request.

## Abbreviations

The following abbreviations are used in this manuscript:

ABC	Artificial bee colony
BAW	Bulk acoustic wave
BVD	Butterworth–Van Dyke
COM	Coupling of modes
FEM	Finite-element method
IDT	Interdigital transducer
LN	Lithium niobate
LT	Lithium tantalate
SAW	Surface acoustic wave
SH	Shear-horizontal
XBAR	Laterally excited bulk acoustic wave resonator

## Appendix A. Results of the Independent ABC Runs

Table A1 lists the individual FEM-based performance metrics used to calculate the statistics in Table 7. Run numbers identify independent runs within each case; corresponding row numbers do not imply paired experiments.

**Table A1:** FEM-based performance metrics from ten independent runs of each case.

Run	Case I IL (dB)	Case I Ripple (dB)	Case I Rejection (dB)	Case II IL (dB)	Case II Ripple (dB)	Case II Rejection (dB)
1	2.24	0.33	40.76	1.76	0.30	40.96
2	1.93	0.36	40.86	1.88	0.37	41.11
3	2.28	0.54	39.39	1.90	0.35	41.39
4	2.37	0.52	44.59	1.83	0.35	41.77
5	2.00	0.43	41.28	1.87	0.40	42.30
6	2.29	0.49	42.06	2.04	0.50	41.18
7	2.55	0.53	43.37	2.07	0.37	41.17
8	2.11	0.46	40.22	1.85	0.31	41.55
9	2.50	0.46	42.43	1.79	0.30	41.07
10	2.56	0.37	42.62	2.05	0.43	41.21

Note: IL denotes worst-case passband insertion loss. All values are in dB and are reported to two decimal places. The optimization settings were fixed within each case, with independent random initialization for each run.
